# Acción PrEP: A Multi‐Method Pilot Design to Assess the Feasibility of Community Co‐Designed Implementation Strategies to Advance PrEP Uptake Among Latines in the U.S. South

**DOI:** 10.1002/jia2.70196

**Published:** 2026-10-08

**Authors:** Sarah M. Wilson, Judith Montenegro, Lisvel A. Matos, Carrie B. Dombeck, Carolina Mejia, Jamilah Taylor, Aleida Espinal, Megan A. Reaves, Cristofer Navas, Samantha Fortier, Reeva Kandel, Amy Corneli

**Affiliations:** ^1^ Department of Psychiatry & Behavioral Sciences Duke University School of Medicine Durham North Carolina USA; ^2^ Department of Population Health Sciences Duke University School of Medicine Durham North Carolina USA; ^3^ Latinos in the South Program Latino Commission on AIDS Durham North Carolina USA; ^4^ Duke University School of Nursing Durham North Carolina USA; ^5^ Duke Office of Clinical Research Duke University School of Medicine Durham North Carolina USA; ^6^ Department of Epidemiology & Biostatistics University of Arizona Tucson Arizona USA; ^7^ Duke Physician Assistant Program Duke University School of Medicine Durham North Carolina USA; ^8^ UNC School of Medicine Chapel Hill North Carolina USA

**Keywords:** health equity, Hispanic/Latino ethnicity, human immunodeficiency virus, implementation science, pre‐exposure prophylaxis, social determinants of health

## Abstract

**Introduction:**

Despite systemic inequities in pre‐exposure prophylaxis (PrEP) uptake among Latines (a gender‐inclusive term for people of Latin American descent, used to promote inclusivity and language justice), clinic‐level implementation strategies addressing health equity‐related PrEP barriers are underutilized. This multi‐method study assessed the feasibility and acceptability of community‐co‐designed, equity‐focused strategies to improve PrEP uptake among Latines in the Southern United States.

**Methods:**

Facilitated by a community‐based organization (CBO)/academic partnership, a workgroup of local community and public health constituents with HIV/PrEP experience from Mecklenburg County, North Carolina, co‐developed PrEP implementation strategies over 6 months (March−August 2023), using implementation mapping, human‐centred design and participatory methods. The resulting strategies were pilot‐tested at a federally qualified health centre and a non‐profit community healthcare organization (March−August 2024). Process measures included quantitative implementation strategy fidelity and descriptive strategy modifications. Outcomes included: quantitative acceptability of the workgroup process (using exit surveys) and qualitative feasibility and acceptability of the implementation strategies (using 23 qualitative key informant interviews conducted October 2024−February 2025).

**Results:**

Ten exit surveys were collected from workgroup members, with 60% identifying as Latine and 40% with HIV lived experience. Members reported high satisfaction with workgroup activities. The workgroup developed three health equity‐focused strategies: (1) a needs assessment; (2) blended implementation facilitation (weekly facilitation meetings between a CBO external facilitator and clinic‐based internal facilitator); and (3) a bilingual, culturally tailored PrEP awareness mass media campaign. Process data indicated: 100% fidelity to the needs assessment, 88% average completion of facilitation meetings and delayed campaign dissemination. Adaptations included fewer facilitation meetings, increased leadership engagement and a sustainability planning workshop. Key informant interviews included six pilot implementers and 17 potential adopters. Notable themes centred on strategy feasibility (e.g. concrete/actionable feedback, staffing challenges impacted feasibility), acceptability (e.g. an approachable implementation team) and perceived strategy benefits (e.g. improved patient outreach and engagement).

**Conclusions:**

The community co‐design process and resulting strategies were generally acceptable and feasible. Study findings highlighted the need to balance rapid actionability with implementation timelines that ensure time for meaningful change. Future trials may test whether this approach to PrEP implementation improves PrEP uptake and sustainment for Latines and other underserved populations.

## 1 Introduction

Despite systemic inequities in pre‐exposure prophylaxis (PrEP) uptake among Latines (a gender‐inclusive term for people of Latin American descent, used to promote inclusivity and language justice) [1, 2], clinic‐level implementation strategies that address health equity‐related PrEP barriers are underutilized [3]. Latines who could benefit from PrEP are less likely than the general population to be prescribed PrEP (14% vs. 23%) [1]. Barriers to successful uptake and persistence include structural (e.g. lack of health insurance), community‐level (e.g. awareness, stigma, cultural/historical trauma) and individual (e.g. inaccessible health information) [4, 5]. PrEP implementation strategies that focus on sociocultural determinants of health and health equity are underutilized in general [6]. Despite calls for greater attention to health equity within implementation science [7], tailored implementation strategies to address sociocultural determinants of PrEP use remain understudied. Failure to address these health equity‐focused PrEP determinants will greatly impede progress towards ending the HIV epidemic.

The Latino Commission on AIDS *Latinos in the South* (LITS) Programme and others emphasize unique HIV‐related experiences of Latines in the Southern United States, including the importance of language access, migration patterns, intersectional stigma and community‐driven solutions [8, 9]. Thus, local communities must be engaged in designing implementation strategies to address vulnerability to HIV among minoritized populations. To mobilize communities to impact public health, engagement science and co‐development are key methodologies [10]. The current study bridged rigorous engagement science with implementation science to test the feasibility and acceptability of community‐co‐developed, equity‐focused strategies to improve PrEP uptake among Latines in the Southern United States.

## 2 Methods

### 2.1 Overall Design and Framework

Following guidance for implementation pilot study designs [11], this study focused on developing and assessing the feasibility and acceptability of a set of community co‐designed implementation strategies. The study's conceptual model drew upon the Exploration, Preparation, Implementation, and Sustainment (EPIS) framework [12], informed by the EPIS Typology of Power in Implementation [13] and the Reconfigured Social Determinants of Health Framework [14]. Key model concepts, such as types of power [13] and the root causes of health inequities [14], were integrated during exploration of implementation determinants and strategies.

### 2.2 Community/Academic Partnership

The study was led by a community/academic partnership. Latinos in the South (LITS) is a community‐based organization (CBO) programme within the Latino Commission on AIDS that focuses on outreach and policy to end the HIV epidemic in the Latine community within Southern states (Alabama, Florida, Georgia, Louisiana, North Carolina, Mississippi, South Carolina, Tennessee and Texas). The academic partner was an HIV‐focused implementation science research team at Duke University School of Medicine.

### 2.3 Study Ethics Approval and Informed Consent

The Duke Health Institutional Review Board determined the project to be exempt from full federal review requirements. All participants who completed qualitative interviews provided verbal informed consent.

### 2.4 Study Team Reflexivity and Positionality

The community/academic team sought to reduce power imbalances [13] in several ways: (1) ensuring equitable funded effort across LITS/Duke teams; (2) making key decisions by consensus; and (3) avoiding scientific jargon during meetings. To minimize bias when making key decisions, team members acknowledged the impact of varying levels of social power and varying lived experiences, such as immigration, first language, racialized identity, sexual orientation and gender identity.

### 2.5 Study Setting

All study activities were focused on access to PrEP in Mecklenburg County, NC, which is one of 48 counties in the United States with the highest number of new HIV diagnoses [15, 16]. The county has seen rapid growth among Latine residents, with current estimates at around 15% [17].

### 2.6 Methods for the Implementation Strategy Co‐Development Process

LITS led recruitment of the Acción PrEP workgroup through existing community networks. Workgroup members were paid study consultants. Members included individuals taking PrEP, individuals living with HIV, public health and healthcare workers, immigration justice advocates and individuals with ties to Latine communities or who work with Latine clients.

LITS and Duke collaboratively co‐facilitated workgroup meetings. Workgroup members completed an optional demographic survey. Meetings were 90 min each and were held monthly for 6 months (March−August 2023) with simultaneous Spanish/English interpretation. Workgroup agendas were developed using the typology of power in EPIS [13], implementation mapping [18] and the *Workshop Tactics* series (a compendium of user‐centred meeting activities) [19].

Workgroup meetings followed these steps: (1) identify adopters and implementers; (2) prioritize equity‐related PrEP implementation determinants; (3) iteratively draft implementation strategies; and (4) finalize implementation strategies. The final set of implementation strategies were categorized by Expert Recommendations for Implementing Change (ERIC) categories [20] and specified using established recommendations [21].

### 2.7 Methods for the Implementation Feasibility Pilot Study

Following implementation strategy creation, the partnered CBO/academic team carried out a 6‐month pilot test of the strategies within two local healthcare organizations (March−August 2024). Healthcare organizations were recruited by LITS and the Acción PrEP workgroup and received $3000 in compensation for participating in the 6‐month pilot, which was meant to partially defray time costs involved in participation.

The healthcare organizations were a federally qualified health centre (FQHC) with clinical offerings addressing social determinants of health (SDOH) and a non‐profit health centre focusing on healthcare for uninsured/underinsured patients. Prior to implementation, both organizations reported that they already provided Spanish‐language services to patients. Additionally, both organizations reported already providing HIV services and PrEP services.

### 2.8 Feasibility and Acceptability Outcomes

#### 2.8.1 Quantitative Workgroup Acceptability Survey

An optional, anonymous quantitative exit survey was used to assess acceptability of the co‐design process among workgroup members (August 2024). Items in the exit survey were adapted from a published measure of group dynamics in community‐engaged research [22].

#### 2.8.2 Qualitative Implementation Strategy Feasibility and Acceptability

Qualitative semi‐structured interviews (October 2024−January 2025) assessed implementation strategy feasibility and acceptability among two groups: (1) staff from the two organizations who completed the implementation pilot (i.e. internal facilitators, leaders and other staff/clinicians involved in PrEP care) and (2) potential adopters (i.e. those not yet exposed to the strategies). Potential adopters were recruited at the LITS annual Encuentro Conference in October 2024 and by word of mouth from partner organizations across the Southeast. Potential adopters were recruited because they could be involved in future implementation efforts. Given LITS’ strong partnerships across the Southeast, Acción PrEP expansion could occur in other locations. The only exclusion criterion was participation in the workgroup, as this could bias responses. Participants were compensated with a $50 gift card. Interviews were completed in English or Spanish.

Implementers were interviewed about their perceptions of feasibility (e.g. how strategies had been carried out), acceptability (e.g. helpfulness and acceptance of the strategies) and appropriateness (e.g. how well suited the strategies were for their setting). Potential adopters were presented with a brief overview of Acción PrEP, then asked their perceptions of feasibility, acceptability and appropriateness for the clinics in their geographic area. Following each interview, the interviewer completed a debriefing form highlighting the main themes, any unique participant perspectives and any interview challenges. Interviews were transcribed by a professional transcription service, Spanish interviews were translated to English by professional translators and all transcripts were quality‐checked for accuracy [23].

#### 2.8.3 Multi‐Method Analysis

For quantitative analysis of workgroup acceptability, descriptive statistics were generated from the workgroup exit survey. Cutoff criteria are not available for this measure, so simple descriptive statistics were calculated (range, mean, standard deviation).

All adaptations that the study team made to the original workgroup‐developed implementation strategies were reported descriptively using the Framework for Reporting Adaptations and Modifications to Evidence‐based Implementation Strategies (FRAME‐IS) [24]. This is a methods framework for documenting modifications to implementation strategies that specifies the content and rationale for each modification.

Applied thematic analysis [25] was used to analyse interviews. The Principal Investigator (PI) and two analysts independently reviewed debriefing forms and noted emergent themes, then met to discuss and agreed on a common set of codes [26]. The analysts next used NVivo 14 qualitative analysis software to code and organize transcripts, applying agreed‐upon codes and adding new codes as necessary. Prior to coding independently, analysts conducted inter‐coder reliability sessions [27], resolving coding discrepancies through discussion, until codes were applied consistently. Following coding, the team met to synthesize codes into themes, reviewing code frequencies and discussing participant narratives to determine salient themes to describe participants’ perspectives. Analysts then drafted memos with thematic content and illustrative quotes, which were reviewed and iteratively refined. Due to differences between experiences of implementers and potential adopters, analyses were stratified and primarily emphasized implementer themes.

## 3 Results

### 3.1 Findings From the Implementation Strategy Co‐Development Process

#### 3.1.1 Acción PrEP Co‐Design Workgroup Characteristics

Among the working group members (*N* = 15), men (60%), women (40%), sexual minorities (60%) and individuals who self‐reported Latine ethnicity (60%) were represented. Nearly all workgroup members spoke English conversationally (93%), and most also spoke Spanish conversationally (73%). A majority of workgroup members worked in healthcare or public health (73%), and most considered themselves to be community advocates (60%). Many workgroup members (40%) reported direct lived experience of HIV (either themselves or a family member) (Table 1).

**TABLE 1:** Summary characteristics of Acción PrEP community co‐design workgroup consultants, *N* = 15.

Variable	*N* (%)
Gender	
Men	9 (60%)
Women	6 (40%)
Sexual orientation	
Gay or same‐gender loving^a^	7 (47%)
Straight or heterosexual	6 (40%)
Bisexual or pansexual	2 (14%)
Race/ethnicity	
Hispanic, Latino/a, Latine or Latinx	9 (60%)
Black or African American	5 (33%)
Native American, Spanish speaker	1 (7%)
Languages spoken conversationally^a^	
Spanish	11 (73%)
English	14 (93%)
Other	0 (0%)
Age	
18−24	1 (7%)
25−34	4 (27%)
35−44	6 (40%)
45−54	2 (13%)
55−64	2 (13%)
Experience/interest in HIV prevention^b^	
Direct lived experience of HIV^c^	6 (40%)
Healthcare worker or public health worker	11 (73%)
Community advocate/activist	9 (60%)

*Note*: This table shows characteristics of the Acción PrEP co‐design workgroup, a group of paid study consultants representing various constituencies within Mecklenburg County, NC, USA. Workgroup members collaborated for 6 months to create a set of co‐designed implementation strategies to improve PrEP uptake among Latines in the Southern United States.

Abbreviation: PrEP, pre‐exposure prophylaxis for HIV.

^a^Please note that the sexual orientation “same‐gender loving” is a culturally specific term from Southern U.S. Black/African‐American culture.

^b^Totals may add up to greater than 100% because respondents could check off more than one option.

^c^Self or family member has direct lived experience of HIV or HIV prevention.

#### 3.1.2 Workgroup Co‐Development Deliverables

Table 2 shows Acción PrEP workgroup activities and deliverables. Workgroup members identified key determinants of equitable PrEP implementation among Latines, which were rated by impact. High‐impact determinants were then categorized as high/medium/low effort to change. Determinants categorized as high‐impact included (but were not limited to): PrEP knowledge; provider readiness to discuss PrEP; patient documentation/immigration status; intersecting stigma; cultural barriers; and systemic language justice. These determinants were then synthesized by the partnered CBO/academic team into discussion questions for solution‐focused brainstorming. This brainstorming led to further implementation strategy development using idea beetles (a workshop activity to rapidly develop idea details) [19].

**TABLE 2:** Acción PrEP community workgroup implementation strategy co‐design activities and deliverables, March through August 2023.

Meeting	Activities	Deliverables
1	Orientation (in‐person): introductions; project overview and goals; discuss members’ hopes and concerns for the workgroup in small groups	**Orienting to the mission** Summarized hopes and concerns for community/academic partnership to take into consideration
2	Strengths and challenges (virtual): empathy‐driven identification of key stakeholders; brainstorming multi‐level (individual, community, system), local Latine community PrEP implementation determinants in small groups	**Community‐defined implementation determinants** Cross‐cutting determinants: knowledge; attitudes; stigma and bias; language; where you are from and where you live Personal‐level: social connections; financial resources/PrEP affordability Community‐level: collaboration and networking; trust, kindness and empathy; inclusion and representation; misinformation and mistrust Health system‐level: clinical resources and non‐provider roles (i.e. peer navigator); CDC PrEP locator tool; eligibility barriers; health policy changes
3	Prioritizing strengths and challenges (in‐person): design thinking principles; workgroup member presentations on lived experience and research; impact voting on determinants; effort‐to‐change discussion on high‐impact determinants; general consensus‐building on key focus areas for implementation strategies	**High‐impact determinants** Low effort to change: PrEP knowledge; eligibility barriers Medium effort to change: system‐wide knowledge; provider readiness to discuss PrEP; patient documentation/immigration status; intersecting stigma (PrEP, HIV and other identities); health staff knowledge, skills and attitudes High effort to change: cultural barriers (machismo and caballerismo); system‐wide bias, stigma and lack of knowledge; systemic language justice
4	Planning actions (virtual): breakout groups to use solution‐focused brainstorm techniques to address high‐impact implementation determinants from meeting 3	**Initial brainstormed solutions** (1) Public awareness campaign that promotes inclusivity across ages and demographics; partnering with community agencies to educate about PrEP accessibility; (2) promoting local outreach; (3) increasing language access for patients through bilingual staff; (4) promoting cultural humility and cultural competency among staff; (5) ensuring forms are in patients’ preferred language; (6) increased access to PrEP navigators; (7) extended clinic hours; (8) reduced patient wait time
5	Planning actions continued (in‐person): small group “Idea Beetle” activity to refine implementation strategies designed to enhance PrEP access within the health system.	**Further refined elements of implementation strategies** (1) Assessments: number of bilingual staff currently employed; clinic documents available in English and Spanish; clinic availability and wait times; patient‐to‐PrEP navigator ratio to ensure adequate support. (2) Implementation Facilitation: cultural humility and competence trainings; increase PrEP navigation; help ensure language access; ensure forms and documents are inclusive; educate providers about PrEP availability; increase readiness to refer patients to PrEP.
6	Finalizing actions (virtual): overview of future actions for ACCION PrEP. Workgroup prioritized strategies into actionable, clinic‐level tasks.	**Action recommendations** (1) PrEP awareness campaign, (2) facilitate revision of clinic documents, (3) clinician trainings on PrEP to increase knowledge, reduce stigma and increase cultural humility, (4) redesign PrEP navigation in clinics, (5) help clinics plan for hiring, retaining and certifying bilingual staff and (6) create an access assessment for each clinic

*Note*: This table shows the activities and deliverables of each meeting of the Acción PrEP co‐design workgroup, a group of paid study consultants representing various constituencies within Mecklenburg County, NC, USA. Workgroup members collaborated during six meetings across 6 months to co‐create a set of implementation strategies to improve PrEP uptake among Latines in the Southern United States.

Abbreviation: PrEP, pre‐exposure prophylaxis for HIV.

This 6‐month iterative co‐design process yielded six discrete actions recommended by the workgroup. These six actions were then condensed into a smaller set of implementation strategies. The workgroup provided final approval for these changes.

The final Acción PrEP Implementation Package consisted of: (1) Access Assessment (a community‐led, multi‐method audit of PrEP accessibility for Latine clients); (2) Community‐Led Blended Facilitation [28] (an external facilitator from LITS paired with a designated internal facilitator from the target clinic); and (3) Culturally Tailored PrEP Awareness Campaign (bilingual materials specifically tailored for the local Latine community). See Table 3 for a full description of implementation strategies and Figure 1 for a visual depiction of implementation determinants and strategies yielded by the workgroup.

**TABLE 3:** Specifications for implementation strategies co‐developed by the Acción PrEP community co‐design workgroup.

Domain [21]	Strategy 1: Clinic PrEP access assessment	Strategy 2: Community‐led blended facilitation	Strategy 3: Culturally tailored PrEP awareness campaign
**ERIC category** [20]	Use evaluative and iterative strategies	Provide interactive assistance	Engage consumers
**Actor(s)**	Community organizer; implementation science academic team	Community organizer; clinic internal facilitator; implementation science academic team	Community organization; clinic internal facilitator; clinic staff
**Action(s)**	Assess accessibility of PrEP for Latine clients through multi‐method community co‐created audit, including clinic website and pamphlet review, clinic space walk‐through, staff informational interview, leadership survey and mystery calls from “secret shoppers.” Provide assessment report to leadership and staff. Use information from assessment to tailor facilitation.	Community organizer serves as external facilitator, meeting regularly with a clinic internal facilitator to review PDSA cycles and problem‐solve barriers. The community organizer receives consultation regularly from the implementation science academic team. Clinic informatics specialists submit numerical data on PrEP patients and prescriptions monthly, so the academic team can prepare visualizations and reports for review by the facilitators. Clinic leadership meet with the external facilitator and academic team as needed.	Community organization leads development of a PrEP awareness campaign specifically tailored for the local Latine community. Marketing focus groups and design considerations are used to create Spanish and English postcards with a QR code and linked website.
**Target(s)**	Clinic leadership; clinic internal facilitator	Clinicians; clinic staff; clinic physical spaces and culture	Community members
**Temporality**	Access assessment should be completed prior to the start of blended facilitation	Facilitation begins immediately following the access assessment	Public awareness campaign begins 3−4 months into the facilitation period.
**Dose**	Once during pre‐implementation, with audit activities lasting approximately 1 month	Weekly facilitation meetings lasting 60 min (in‐person or virtual as preferred); meetings occur over a 6‐month period	One shipment of postcards to each clinic was planned for feasibility and acceptability testing.
**Implementation outcome(s) affected**	Implementation readiness	Provider PrEP adoption; Latine patient PrEP uptake	Latine patient PrEP uptake
**Justification**	In co‐design sessions, the Acción PrEP workgroup identified the importance of fully understanding each clinic's unique context, strengths and barriers before attempting any changes in the clinic.	Co‐design sessions underscored the complexity of PrEP access among Latines. The workgroup theorized that multiple tailored, synergistic changes are necessary to improve PrEP access.	The Acción PrEP workgroup specifically underscored the impact of low community knowledge about PrEP and financial resources for PrEP visits and prescriptions.

*Note*: This table shows the specifications for the implementation strategies co‐created by the Acción PrEP co‐design workgroup, a group of paid study consultants representing various constituencies within Mecklenburg County, NC, USA. Workgroup members co‐created a set of implementation strategies to improve PrEP uptake among Latines in the Southern United States. The co‐designed implementation strategies are specified per guidance from Proctor et al. [21].

Abbreviations: ERIC, Expert Recommendations for Implementing Change [20]; PrEP, pre‐exposure prophylaxis for HIV.

**FIGURE 1:**
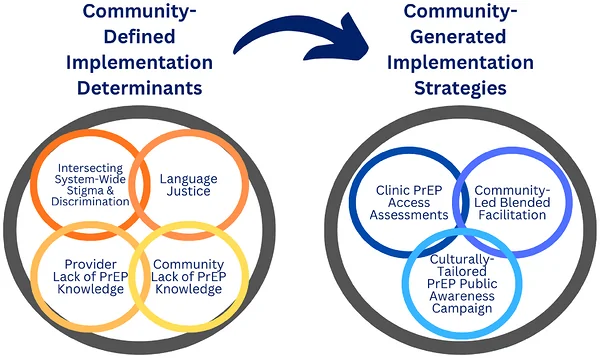
Community co‐created model of implementation determinants and implementation strategies co‐developed by the Acción PrEP community co‐design workgroup during meetings. *Note*: This figure shows implementation determinants (i.e. the factors that influence uptake of PrEP among Latines in the Southern United States) co‐developed by the Acción PrEP co‐design workgroup across meetings 2 through 6. After development and prioritization of implementation determinants (meetings 2 and 3), the workgroup also generated implementation strategies based on these determinants (meetings 4–6). See Table 2 for specific activities that led to these determinants and strategies. See Table 3 for specifications of the implementation strategies depicted in this figure. Abbreviation: PrEP, pre‐exposure prophylaxis for HIV.

#### 3.1.3 Workgroup Acceptability Outcomes

In the optional workgroup exit survey (*N* = 10), acceptability was rated highly, as were comfort expressing their opinions, increases in social/professional networks and knowledge, and the capacity of the workgroup to influence decisions that affect the community (Table 4). All workgroup members somewhat or strongly agreed that the workgroup worked together to solve problems and was effective in achieving its goals.

**TABLE 4:** Self‐reported, post‐design process satisfaction ratings from Acción PrEP community co‐design workgroup consultants, *N* = 10.

Dimension of satisfaction	Possible range^a^	Observed range	M (SD)
Comfort in expressing your opinion during workgroup meetings	1−5	4−5	4.8 (0.42)
How well the group worked together to solve problems	1−5	4−5	4.4 (0.52)
Overall satisfaction with activities of the workgroup	1−5	2−5	4.6 (0.97)
Overall satisfaction with the progress of the workgroup	1−5	3−5	4.7 (0.67)
Your opinion was listened to and considered by others in the workgroup	1−5	4−5	4.6 (0.52)
Increased social or professional network	1−5	3−5	4.6 (0.70)
Increased knowledge about important topics	1−5	4−5	4.8 (0.44)
Workgroup can influence decisions that affect the community	1−5	4−5	4.8 (0.42)
Workgroup was effective in achieving its goals	1−5	4−5	4.5 (0.53)

*Note*: This table shows self‐reported satisfaction scores completed by the Acción PrEP community co‐design workgroup, relating to their experience during the co‐design process. The workgroup consisted of paid study consultants representing various constituencies within Mecklenburg County, NC, USA. Workgroup members collaborated for 6 months to create a set of co‐designed implementation strategies to improve PrEP uptake among Latines in the Southern United States. Self‐reported satisfaction was completed anonymously and voluntarily at the end of the co‐design process.

Abbreviation: M, mean; PrEP, pre‐exposure prophylaxis for HIV; SD, standard deviation.

^a^All items were scored on a 5‐point Likert scale with higher scores indicating higher levels of agreement.

### 3.2 Findings From the Implementation Pilot

#### 3.2.1 Characteristics of Implementing Organizations

Both implementing organizations provided the study team with quality improvement information pulled by their own staff on patient populations at baseline (see Appendix 1). In the month prior to implementation, the FQHC reported 806 patients seen by 14 clinicians across two clinics and recorded nine PrEP prescriptions. The non‐profit health centre reported 1788 patients seen by 13 clinicians across two clinics and recorded 34 PrEP prescriptions in the month prior to implementation.

#### 3.2.2 Implementation Strategy Adaptations

Three key adaptations were made during the implementation period; Table 5 provides a full description and specifications of all adaptations. Briefly, adaptations involved: (1) shortening the overall duration of facilitation from 6 to 4 months (which also yielded fewer weekly meetings); (2) inviting leadership to attend several facilitation meetings; and (3) hosting a sustainability planning workshop with each organization.

**TABLE 5:** Implementation strategy adaptations completed by the Acción PrEP community/academic partnership study team.

Modification	Rationale	What	Nature of the change relationship to core elements	Goal of modification	Level of modification
Shorten overall duration of blended facilitation from 6 to 4 months	Ensure timely pilot completion within 6‐month implementation period	Context— other (duration)	Shortening/condensing Core elements of the strategy were maintained	Increase feasibility of implementation	Implementer level
Include leadership in 3−4 blended facilitation meetings	Increase leadership buy‐in and internal facilitator power to make process or cultural changes in the organization	Context—personnel	Integrating another strategy Core elements of the strategy were maintained	Increase feasibility of implementation	Implementer level
Sustainability planning workshop 2 months after blended facilitation ended	Increase the likelihood that change efforts will continue	Content	Adding elements Core elements of other strategies were maintained	Improve sustainability of implementation	Organizational level

*Note*: After implementation strategies were created by the Acción PrEP community co‐design workgroup, the community/academic partnership study team (academic partner Duke and community partner Latinos in the South) carried out the strategies (March−August 2024). During this time, adaptations to the originally co‐designed strategies were made by the study team. This table shows the adaptations, which are reported consistent with recommendations from Framework for Reporting Adaptations and Modifications to Evidence‐based Implementation Strategies (FRAME‐IS) [24].

Abbreviation: PrEP, pre‐exposure prophylaxis for HIV.

#### 3.2.3 Implementation Process and Fidelity

All Access Assessment components were completed by the CBO/academic partnership team at both healthcare organizations with 100% fidelity. Both organizations completed a feedback and planning meeting where the organization's leadership and internal facilitator reviewed the Access Assessment. Within each Access Assessment, organizational strengths (i.e. strong leadership, staff customer service and passion for HIV advocacy), areas for change (i.e. more quickly engaging new PrEP patients, consistent PrEP referral and follow‐up, and intersectional inclusivity) and recommended change strategies (i.e. revise process for new PrEP patients, PrEP training and reminder cards for staff, equity‐focused listening sessions, and signs and symbols of a welcoming environment for LGBTQ+ Latines and Spanish‐speaking patients) were communicated.

Blended facilitation meetings began in late April/early May 2024 and proceeded for 16 weeks through late August 2024. Both organizations had high fidelity to weekly facilitation meetings. At the non‐profit healthcare centre, 15 of the 16 planned weekly blended facilitation meetings occurred (93.8%). At the FQHC, 13 of the 16 planned weekly meetings occurred (81.3%). Overall, 88% of allocated weekly slots for blended facilitation occurred.

Deployment of the public awareness campaign was delayed. Originally planned for mid‐summer, the campaign materials were sent to facilities in late August 2024, in time to distribute at the local municipal LGBTQ+ Pride festival. Each facility received approximately 500 postcards, with a tailored organization logo and contact information. Organizations reported successful distribution of postcards.

#### 3.2.4 Qualitative Interview Participant Characteristics

Organizational leaders nominated prospective participants who were meaningfully involved in implementation and able to provide feedback on the process. Given the small size of the two organizations, there were few staff members who were involved in implementation efforts and would be able to comment on the implementation strategies and process. Across the two implementing organizations that conducted implementation strategies, eight staff members were invited to participate (four from each organization).

After outreach for interview participation, two staff members were considered lost to contact after four requests to participate in an interview. Among the eight invited, six staff from the implementing healthcare organizations (*n* = 2 from the FQHC and *n* = 4 from the non‐profit health centre) participated. Implementer staff roles overall that were represented across the two facilities included assigned site internal Acción PrEP implementation facilitator, facility lead HIV clinician, chief medical officer, director of clinical operations and business administrator. Additionally, 17 potential adopters participated in interviews. Overall, 19 interviews were conducted in English, and four were completed in Spanish.

### 3.3 Qualitative Themes: Implementer Perceptions of Acción PrEP Feasibility

#### 3.3.1 Concrete, Actionable Feedback

Implementers (*n* = 6) generally found the Acción PrEP Access Assessment to be concrete and actionable. Implementers appreciated that the Access Assessment allowed engagement with their clinic team in implementing change, and some noted that the Access Assessment's goals enabled change. For example, one implementer said, “*The Acción PrEP implementation has really helped to kind of get everyone on the same page and streamline a specific workflow. […] And this really helped out to kind of get everyone on the same page of what we need, how we wanna look, and how we wanna present ourselves to all these patients*.” (Participant 1)

Other implementers noted the importance of receiving resources from the study team and that the concrete, actionable nature of change goals led to noticeable improvements in clinic processes and patient outcomes (*n* = 2).

#### 3.3.2 Staffing Challenges Impacted Feasibility

All implementers (*n* = 6) described experiencing one or more barriers that impacted feasibility during implementation. Implementers’ challenges were primarily related to staffing (*n* = 6). They reported that teams had difficulty balancing clinic demands, limited staffing and maintaining Acción PrEP change efforts, with some saying the strategies were too time‐consuming given other responsibilities. As one implementer explained,

“*I'd say the challenges that we had were more so ‘cause we were short‐staffed during a lot of this*. […] *It's very time‐consuming and trying to get things done as well as do your regular job. So, I think that created a problem, just that kind of timing like that. And then some of the meetings were just a little bit challenging to get it done. And then the other thing is our window of working on this project was very small. So, there were a lot of things where you had to go and get it done now versus three months, three or four months versus a year or so to work on a project*.” (Participant 2)

#### 3.3.3 Internal Facilitator Support Challenges

Most implementers (*n* = 5) described challenges supporting the designated internal facilitator, including difficulty maintaining protected time and issues with the internal facilitator not having sufficient familiarity with clinic workflows. Facilitators were often busy with other clinic duties, so driving change efforts was challenging. As one implementer stated, “*[The facilitator role] was very unfeasible. I wish I had some more time. This was one of the projects, like I told you earlier, like I enjoyed it and I loved it, and I was excited. […] It's just I wasn't able to sit down and actually take my time. I felt rushed all the time*.” (Participant 3) Additionally, in some cases, the internal facilitator was perceived as having insufficient awareness of workflows for PrEP prescribing. For example, one implementer stated, “*The barriers they experienced were mostly just because [the internal facilitator] doesn't always understand what the rest of the clinic's doing, which can be remedied […] with just a partnership. You know, two champions versus just one at two different levels in the organization*.” (Participant 4)

#### 3.3.4 Leadership Engagement

Many implementers noted challenges linked to limited leadership receptivity or engagement in the change process (*n* = 4), which made it harder to carry out Acción PrEP implementation strategies within the clinic.

### 3.4 Qualitative Themes: Implementer Perceptions of Acción PrEP Acceptability

#### 3.4.1 Approachable Implementation Team

Nearly all implementers (*n* = 5) said it was easy to work with the Acción PrEP community/academic partnered team, describing the team as “comfortable” and “approachable.” Implementers appreciated the team's supportive nature and willingness to provide additional resources, information or guidance to clinic staff, particularly with respect to the clinic's internal facilitator. As one implementer stated,

“*I think one of the things that was actually really nice about this particular grant, having done other studies like this, is [CBO external facilitator name]. […] She worked very closely with our project [internal facilitator], and they would meet frequently to sort of refine implementation, and refine different things. And that was really helpful, because she was able to give us a direct perspective from the grant team that we maybe wouldn't have had otherwise*.” (Participant 5)

#### 3.4.2 Internal Clinic Role Was Helpful

All implementers (*n* = 6) also regarded the internal facilitator role as helpful. Implementers valued various aspects of the role, including that the internal facilitator was experienced with the clinic and population served and influenced positive outcomes for their clinic and patients. Internal facilitators with contextual experience were perceived as being able to start implementation more quickly and with more autonomy. As one implementer noted:

“*Well, I would say the [Internal facilitator], with her having been in this field for so long and having already had the experience with the research and implementation, I think she was the perfect person to just jump right in and just start running with it. She's been doing this for so long, so she knows about process improvement, those kinds of things*.” (Participant 2)

#### 3.4.3 Strategies Aligned with Clinic Goals and Processes

Half of implementers (*n* = 3) valued the implementation strategies because they aligned with organizational goals. One implementer expressed that this alignment facilitated their clinic's capacity to quickly implement changes: “*[It fit] very well. It all kind of, I like to say, fit like a glove. […] Having well thought‐out action steps helped us to implement that quickly. I believe if not done right, we probably could have taken longer. But it was helpful enough that we were all able to kind of streamline a new process quickly*.” (Participant 1)

Half of implementers also valued that Acción PrEP fit with the clinic's existing desire for oversight to improve clinic processes, explaining that external oversight and training was something their clinic was already interested in having implemented to improve clinic services. As one implementer stated, “*I think it fits really well. […] I think the clinical team has a tendency to be stuck in a very narrow perspective, and anything like this that kind of shakes them out of that and forces them to question their assumptions and presumptions is always helpful. And to make things more accessible for the community, we're trying to decrease the barriers*.” (Participant 4)

### 3.5 Qualitative Themes: Implementer Perceptions of Acción PrEP Benefits and Outcomes

All implementers (*n* = 6) noted positive impacts or benefit of the Acción PrEP implementation strategies, including better outreach and engagement with patients (*n* = 6), improved customer service (*n* = 5), more cultural knowledge and awareness among clinic staff (*n* = 5), improved patient care services (*n* = 4), and greater PrEP knowledge and awareness (*n* = 4).

#### 3.5.1 Patient Outreach and Engagement

Implementers generally believed that Acción PrEP improved patient outreach and engagement in their clinics. One explained that even staff within the Latine community benefited from these trainings, as it helped them identify their own biases that may have impacted patient engagement, inspiring them to be more intentional with outreach.

#### 3.5.2 Customer Service

Implementers largely attributed improvements in customer service to Acción PrEP efforts such as the mystery calls from “secret shoppers,” which were part of the initial Access Assessment. These secret shopper calls represented an avenue for review or feedback that they otherwise would not have had. One implementer explained that the calls improved customer service because they identified actionable gaps in staff performance. This implementer stated,

“*I really love the mystery calls. […]We started doing our own secret shopper calls because we've found out a lot. We found out some employees were really, really good and some team members were not that great at their jobs. That was just basic information. So, it's not only that information because now we're doing secret shoppers for every office and just randomly. So, I think that was very beneficial because I never thought about that, to be honest with you*.” (Participant 2)

#### 3.5.3 Culturally Responsive Care

Some implementers believed the implementation strategies increased cultural, sexual orientation and gender awareness among staff, noting that mindfulness around Latine patients’ needs increased and became habituated. Enhanced cultural awareness improved patient interactions, increasing patient comfort and recognition of PrEP uptake barriers. As one implementer said, “*It just made me more aware of what the barriers are for Spanish‐speaking patients, and what they face when they're seeking PrEP. So, I tried to be more inclusive, and less exclusive*.” (Participant 5)

Staff's increased interest in improving their own awareness or knowledge was also perceived as a benefit of Acción PrEP, including staff interest in more training and guidance around culturally sensitive terminology, open dialogue about their own limitations around cultural awareness and expressed desires to improve.

#### 3.5.4 Staff Awareness of and Comfort With PrEP

Implementers noted increases in PrEP interest and uptake beyond the Latine community as an indicator of improved clinic services and broader community awareness. Some noted that increased staff awareness of PrEP led to improved comfort discussing it with patients. Reminders of PrEP initiatives, processes and talking points helped to refocus attention on the PrEP population and reduced confusion for staff and patients alike.

### 3.6 Qualitative Themes: Potential Adopter Perceptions of Acción PrEP Acceptability and Feasibility

#### 3.6.1 Clinics Would be Receptive

Among potential adopters (*n* = 17), themes largely resembled those from interviews with implementers. Potential adopters overwhelmingly indicated that clinics in their area would be receptive to the Acción PrEP implementation strategies. Several potential adopters emphasized that clinics in their area would likely be open to working with the external facilitation team because of their shared commitment to helping the community. They explained that most organizations involved with HIV prevention and treatment have common goals and that people who work in these organizations supported efforts that enabled them “*to be part of serving the community the best they can*.”

#### 3.6.2 More Clarity Would Enhance Acceptability

Several potential adopters also suggested that clinics’ willingness to use Acción PrEP would be enhanced by clearly presenting the process, its benefits and expectations. A participant explained that to get clinic buy‐in, a presentation should “*start with their whys. Why would you want to come to my clinic?*” and build from there, including clearly laying out the end goals and outcomes that a clinic could expect to see. A few potential adopters suggested that prospective clinics would be interested in hearing about the findings from the Acción PrEP pilot study, as they considered whether to partner with the CBO/academic partnered team.

#### 3.6.3 Staffing‐Related Barriers Could Impede Implementation

Potential adopters also shared several staff‐related barriers that could potentially impede implementation, such as language or cultural barriers, insufficient staff time and training, and resistance to change. The predominant staff‐related barrier, mentioned by all potential adopters, was language and cultural barriers. Potential adopters noted that for clients who speak Spanish or another Latin American regional dialect, clinics often lack sufficient language access. When explaining why clinics do not have sufficient bilingual staff, participants cited key reasons as cost, de‐prioritization of the Spanish‐speaking community, and even, in some cases, reluctance to accommodate Spanish‐language access needs.

### 3.7 Qualitative Themes: Potential Adopter Recommendations for Acción PrEP

#### 3.7.1 Protected Time for Change Efforts

Potential adopters confirmed the importance of protected time for the clinic internal facilitator to carry out their responsibilities, a finding which had also emerged from the implementer interviews. Potential adopters explained that having protected time set aside for the internal facilitator to work on the Acción PrEP change process would serve as a clear sign to other staff that leadership supported the effort. Some participants also pointed to clinic staffing levels and support from leadership as important determinants of whether it would be feasible for the internal facilitator to have protected time to carry out their role. Partners commented that it would be more challenging for the internal facilitator to devote time to the project if they had also been tasked with additional clinic duties due to short‐staffing.

## 4 Discussion

This study is among the first to pilot community co‐design of implementation strategies addressing community‐ and clinic‐level determinants of PrEP uptake among Latines living in the U.S. South. This study is unique since it rigorously assessed feasibility and acceptability of both the co‐design process and resulting implementation strategies. Findings indicated that both were acceptable and feasible.

The co‐design process shares some similarities with prior research. A partnered community/academic team developed implementation strategies to improve PrEP uptake among Latines in New York City [29]. Additionally, a Chicago‐based study focused on using crowdsourcing to design implementation strategies for long‐acting injectable cabotegravir (a newly available form of PrEP) among Black and Latino sexual minority men [30]. Both studies, like the current study, included community collaboration and partnership. However, Acción PrEP is distinct in its shortened 6‐month co‐design process, empowerment of a CBO to enact change locally and emphasis on more rapid actionability through pilot testing. All study activities in Acción PrEP were driven collaboratively through shared resources between community and academic partners, rather than depending primarily on a research team to move the project forward.

Study findings highlighted promising PrEP implementation strategies. First, both implementers and potential adopters were highly open to blended facilitation [28], which to date has not been tested rigorously for promoting PrEP uptake. Second, use of community partners as external facilitators has broad implications for community‐engaged implementation research. Third, the community‐led audit and feedback strategy (Access Assessment) was highly acceptable, with interview participants noting their eagerness for feedback and improvement recommendations. While it is relatively rare for participatory approaches in HIV‐related research to include community partner involvement at all phases of the study [31], the current study engaged community partners to create and carry out actionable change strategies and hands‐on implementation facilitation. Finally, acceptability for co‐design and implementation was high despite the accelerated timeline.

Sociopolitical context and cultural differences may impact the generalizability of this study's findings. Methods for co‐developing implementation strategies are highly transferable to other states or EHE regions within the United States and to international settings, as demonstrated by other recent work in this area (e.g. human‐centred design to optimize cognitive behavioural therapy implementation in Kenya [32]). However, given the contextual specificity of implementation strategies co‐developed during this study, adaptation may be needed for their use outside of the Southern U.S. context, for areas with more established, long‐standing Latine populations (e.g. Florida, Texas), or for different minoritized groups.

Since the study ended, there have been significant sociopolitical events that have altered the implementation context. In November 2025, just a year after this study ended, increased immigration detentions and deportations led to reduced medical care engagement among many Latines in the city where the study took place [33]. This type of event, along with national‐ and state‐level changes to public health funding, is termed a “critical incident” in implementation science (a large‐scale event that would or could alter or interrupt implementation efforts or care delivery) [34]. It is unclear whether the co‐developed strategies will require additional adaptation given the changing outer context.

The current study has limitations. Given the focus on acceptability and feasibility, objective measures of PrEP prescriptions were not rigorously collected. Although participating healthcare organizations submitted aggregated numbers of PrEP patients and prescriptions monthly, these reports were not sufficiently reliable to include as outcome measures. Additionally, the brief study timeline precluded sustainment evaluation, and qualitative interviews did not include questions related to perceived sustainability. Additionally, although the qualitative sample of potential adopters was sufficient (*n* = 17), the low number of implementers (*n* = 6) reduced qualitative information power. However, information power was also buoyed by the study's narrow aim, sampling specificity and strong interview dialogue [35]. Finally, the workgroup included consultants knowledgeable about PrEP, which potentially limited perspectives from those less familiar or hesitant. Inclusion of those individuals could have yielded additional strategies.

## 5 Conclusions

The community co‐design process was acceptable to workgroup members, and the equity‐focused Acción PrEP implementation strategies (carried out with leadership from a CBO) were acceptable and feasible to healthcare staff, as well as potential adopters. Key determinants of success included time and resources to accomplish changes, staff and leadership awareness of and receptiveness to clinic changes, and language access and cultural barriers. Findings underscore that implementation science teams can meaningfully engage and share power with those most impacted by HIV, including those experiencing immigration‐related stressors, Latino sexual minority men and individuals with limited English proficiency. Findings also highlighted that implementation timelines must balance rapid actionability with sufficient duration for meaningful change. Future fully powered trials may test whether this approach to PrEP implementation improves uptake and sustainment for Latines and other underserved populations.

## Author Contributions

**Sarah M. Wilson**: conceptualization (lead), funding acquisition (lead), investigation (equal), methodology (equal), writing – original draft (lead). **Judith Montenegro**: conceptualization (supporting), investigation (equal), project administration (equal), supervision (equal), writing –review and editing (supporting). **Lisvel A. Matos**: investigation (equal), methodology (supporting), visualization (lead), writing – original draft (supporting). **Carrie B. Dombeck**: Formal analysis (lead), investigation (equal), methodology (equal), writing – original draft (supporting), writing – review and editing (supporting). **Carolina Mejia**: investigation (equal), methodology (equal), writing – review and editing (supporting). **Jamilah Taylor**: formal analysis (supporting), writing – review and editing (supporting). **Aleida Espinal**: investigation (equal), writing – review and editing (supporting). **Megan A. Reaves**: data curation (lead), project administration (lead), writing – review and editing (supporting). **Cristofer Navas**: investigation (supporting), methodology (supporting), writing – review and editing (supporting). **Samantha Fortier**: formal analysis (supporting), investigation (supporting), methodology (supporting), writing – review and editing (supporting). **Reeva Kandel**: investigation (supporting), methodology (supporting), project administration (supporting), writing – review and editing (supporting). **Amy Corneli**: Formal analysis (supporting), methodology (equal).

## Funding

Research reported in this manuscript was funded during 1 July 2022 ‐ 30 June 2025 by an Ending the HIV Epidemic research supplement to the Duke University Center for AIDS Research (CFAR), an NIH‑funded program (Award Number 5P30 AI064518‑18). The content of this article is solely the responsibility of the authors and does not necessarily represent the official views of the National Institutes of Health.

## Conflicts of Interest

The authors declare no conflicts of interest.

## Supporting information




**Appendix 1**: Clinic characteristics within implementing organizations 1 month prior to implementation period (February 2024).

## Data Availability

Study data are available from the study team upon request.
