# Supplementary material for: Acción PrEP: A Multi‐Method Pilot Design to Assess the Feasibility of Community Co‐Designed Implementation Strategies to Advance PrEP Uptake Among Latines in the U.S. South
Source: J Int AIDS Soc. 2026 Oct 8;29(Suppl 5):e70196. doi: 10.1002/jia2.70196 (PMC13650395; doi:10.1002/jia2.70196)
Supplement: Supplementary file 1 [file JIA2-29-e70196-s001.docx]

Appendix 1. Clinic Characteristics within Implementing Organizations One Month Prior to Implementation Period (February, 2024).

|  | **FQHC** | | **Non-Profit Health Center** | |
| --- | --- | --- | --- | --- |
|  | **FQHC Clinic 1** | **FQHC Clinic 2** | **Non-Profit Clinic 1** | **Non-Profit Clinic 2** |
| **Number of Patients Seen**  **N** | 345 | 461 | 415 | 1373 |
| **Hispanic/Latine Patients**  **N (% of Total)** | 195 (56%) | 319 (69%) | Estimate unreliable | Estimate unreliable |
| **Number of PrEP Prescriptions**  **N** | 7 | 2 | 25 | 9 |
| **Number of Clinicians**  **N** | 7 | 7 | 7 | 6 |
| **Clinicians Who Prescribed PrEP**  **N (% of Total)** | 3 (43%) | 2 (29%) | 7 (100%) | 6 (100%) |

Table Legend: This table shows characteristics of the clinics that were selected for implementation within implementing organizations. One implementing healthcare organization was a federally qualified health center (FQHC), and the other was a non-profit health center. Clinic characteristics were derived from aggregated quality improvement data sent to the study team by participating clinics. Please note that these numbers were provided by the implementing organizations and were not verifiable by the research study team. Leaders at the non-profit health center noted some concerns regarding validity and reliability of their data pulls on the number of Hispanic/Latine patients seen in their clinics. Thus, the estimate sent by their staff are not shown in this table.
